# Distorted Building Image Matching with Automatic Viewpoint Rectification and Fusion

**DOI:** 10.3390/s19235205

**Published:** 2019-11-27

**Authors:** Linwei Yue, Hongjie Li, Xianwei Zheng

**Affiliations:** 1 School of Geography and Information Engineering, China University of Geosciences, Wuhan 430074, China; yuelw@cug.edu.cn; 2 State Key Laboratory of Information Engineering in Surveying, Mapping and Remote Sensing (LIESMARS), Wuhan University, Wuhan 430079, China; lihongjie@whu.edu.cn

**Keywords:** building image matching, repetitive structure, transform invariant low-rank textures (TILT), grid-based motion statistics, viewpoint fusion

## Abstract

Building image-matching plays a critical role in the urban applications. However, finding reliable and sufficient feature correspondences between the real-world urban building images that were captured in widely separate views are still challenging. In this paper, we propose a distorted image matching method combining the idea of viewpoint rectification and fusion. Firstly, the distorted images are rectified to the standard view with the transform invariant low-rank textures (TILT) algorithm. A local symmetry feature graph is extracted from the building images, followed by multi-level clustering using the mean shift algorithm, to automatically detect the low-rank texture region. After the viewpoint rectification, the Oriented FAST and Rotated BRIEF (ORB) feature is used to match the images. The grid-based motion statistics (GMS) and RANSAC techniques are introduced to remove the outliers and preserve the correct matching points to deal with the mismatched pairs. Finally, the matching results for the rectified views are projected to the original viewpoint space, and the matches before and after distortion rectification are fused to further determine the final matches. The experimental results show that both the number of matching pairs and the matching precision for the distorted building images can be significantly improved while using the proposed method.

## 1. Introduction

Huge volumes of urban scene images that contain urban buildings are now easily accessible with the rapid development of new sensors. Establishing feature correspondences between the building images is essential to a wide array of applications, including image georeferencing, urban three-dimensional (3D) modeling, visual localization, scene recognition, and augmented reality (AR) modeling [1,2,3,4]. However, the building images usually come from multiple sources, e.g., the street-view images that were captured by low-cost consumer-level cameras or smartphones, in freely varying positions and viewpoints. Therefore, the available building images in the urban environment generally suffer from large projective distortions, especially large perspective deformations, which brings extra difficulty to wide-baseline image matching [5,6]. 

**Figure 1:**
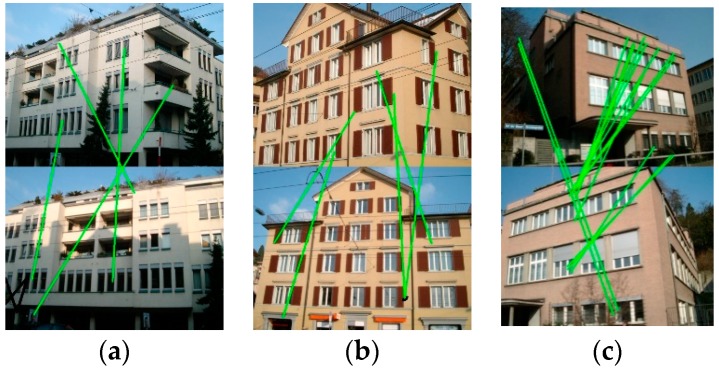
The results of scale-invariant feature transform (SIFT) feature matching in wide-baseline scenarios. (**a**)–(**c**) are SIFT matching results of building image pairs with different view angles.

The main concept of image matching is to find the correspondences between the image pairs for the same scene. This has been a research hotspot for decades, and numerous feature detection and image matching methods have been proposed [7,8,9,10]. Many works have been presented for matching images with different viewing angles. For example, the Harris affine detector and Hessian affine detector are two feature detectors that are based on affine normalization, which are invariant to scale and rotation [11,12]. Moreover, the maximally stable extremal regions (MSER) detector that was proposed by Donoser and Bischof [13] has further enhanced the performance of wide-baseline matching. Furthermore, the classical local feature detectors, e.g., histogram of oriented gradients (HOG) [14], were designed to adapt to image scale and rotation transformations, and they have shown good performances in human detection. A notable milestone was the scale-invariant feature transform (SIFT) detector [15], which is invariant to scale and rotation, and is partially invariant to viewpoint and illumination changes. The speeded-up robust features (SURF) method further promotes the computation efficiency of SIFT without sacrificing the matching accuracy [16]. However, all of these methods are not fully affine invariant, and they are prone to failing when matching image scenarios with widely separate views, as shown in Figure 1. In these cases, the significantly different viewing angles can cause a low matching similarity for images of the same building, which thus leads to sparse and incorrect matches. Inspired by the local-invariant based methods, some researchers have strived to develop more robust feature detectors for image matching with large projective distortions [17]. Typical examples, such as affine-SIFT (ASIFT) [18] and perspective-SIFT (PSIFT) [19], simulate views from different perspectives with affine or perspective transformations before matching, and thus extract features at varied view angles. There are also matching methods that are based on the combination of Scott’s proximity matrix and SIFT features [20]. These methods are robust in dealing with image scenarios with similarity or affine transformations, as well as little noise and illumination changes. However, finding feature correspondences between building images with large perspective deformations that are caused by the large variations in viewpoints and scales remains a challenging task.

Instead of trying to seek the local-invariant features of the images, researchers have also proposed other solutions to the problem of image matching with viewpoint variations. Some of the researchers have tried to correct the differences in view angle to allow for matching while using classical local features. One category of methods is based on the camera pose and 3D geometric information (e.g., stereo models [21], depth maps [22], and 3D sparse meshes [23]) to generate synthetic images that are approximately aligned to the viewpoint of the target image, and thus realize view-dependent SIFT matching. However, these methods require the reconstruction of the dense 3D geometry for the images, and they are strongly restricted by the quality of the reconstructed 3D information. While considering the regular characteristics of building facades, some researchers have explicitly treated the facade as a near-regular texture and they have attempted to match unique tiles representing the pattern element. Doubek et al. [24] matched the similarity of repetitive patterns by comparing the grayscale tiles, the peaks in the color histogram, and the sizes of the two lattices. Bansal et al. [25] studied both urban street-level images and airborne images with extreme viewpoint and illumination differences. Afterwards, they proposed a scale-selective self-similarity descriptor to recognize and segment the structure of facades, and matching was then implemented with a Bayesian classification framework. Wolff et al. [26] analyzed the lattice structures of urban scene facades, and combined the color, shape, and edge-based features to deduce the regularity of the facade pattern for the problem of aerial to street-view facade matching. This category of methods mainly relies on the detection of the regularity of building facades. However, the matching precision is significantly decreased when the quality of the detected structure is influenced by the extreme viewpoint differences. 

With the great success of deep learning methods applied on two-dimensional (2D) images, a recent trend is to perform the image matching process through training a deep convolutional neural network (DCNN). The deep learning based methods have shown good performances in feature point detection and orientation estimation [27,28,29,30]. Deep networks have been used to learn covariant feature detectors in terms of feature point detection [31], particularly toward invariance against affine transformations. For the estimation of orientation, Yi et al. [30] used a Siamese network to predict the orientations that minimized the distance between the orientation-dependent descriptors of matching key points, assuming that the key points had been extracted while using some other technique. Han et al. [29] proposed MatchNet for training a Siamese CNN for general wide-baseline viewpoint invariant matching, followed by a fully connected network to learn the comparison metric. Moreover, Yi et al. [28] introduced a deep network architecture, i.e., learned invariant feature transform (LIFT), to implement the full feature point handling pipeline (detection, orientation estimation, and feature description) in a unified manner, while also preserving end-to end differentiability. However, the previous deep learning based methods usually have a high error rate when estimating the homography for the wide-baseline images with large distortion. Furthermore, the dataset construction for training a deep network is time-consuming. The effectiveness of the trained deep network may be compromised if the viewpoint differences between the matching pairs are out of the scope of the sample datasets.

The distortions can also be corrected with viewpoint normalization or rectification, where the distorted images are rectified to standard views. Cao et al. [32] proposed normalizing the scene planes by identifying the projections of the parallel linear features in the urban building images, and the viewpoint invariant features were then extracted from the rectified views of building facades. The main problem here is that the extraction of accurate line or panel features of the building facades and multi-plane segmentation for the 3D building layout are both necessary, which is highly complex and can be influenced by noise, illumination changes, and geometric distortions [33]. Zhang et al. [34] used transform invariant low-rank textures (TILT) [35] to rectify the distorted images before matching the feature correspondences. In their work, they proposed detecting the low-rank texture regions based on the extracted parallel linear features. As a result, the proposed method was confronted with similar limitations to the methods based on 1D line feature extraction. Moreover, the 3D geometric structure of the building scenes was not fully considered in the process of low-rank texture region extraction, which led to failure in the cases with more complex layouts, e.g., multi-plane building walls. In fact, the TILT algorithm uses convex optimization techniques from matrix rank minimization to extract geometrically and texturally invariant features in a ‘global’ way (as compared with the local feature detectors) [35], and it does not require the incorporation of 3D geometry priors or low-level features, such as corners or edges.

In this paper, based on the above analysis, we propose a method for the wide-baseline image matching of distorted urban building images, based on automatic viewpoint rectification and fusion. The TILT algorithm implements the image rectification, which works effectively and robustly for a wide range of symmetric patterns and regular structures in real scenes, even in the case of significant corruption and warping [35,36]. However, TILT needs a user-specified window to identify the candidate low-rank regions in an image, which is not practical in real applications [35]. Differing from the previous works, we propose an automatic and efficient low-rank texture region detection method through the use of multi-level clustering for the detected local symmetric features [37], thus avoiding the costly high-level structure detection on the building facades and multi-plane segmentation. After viewpoint rectification while using the TILT algorithm, the robust Oriented FAST and Rotated BRIEF (ORB) feature detector is used to match the images [38], for both the original and rectified views. Afterwards, the grid-based motion statistics (GMS) and random sample consensus (RANSAC) techniques are introduced to eliminate the incorrect matches [39,40]. Finally, we project the matching results that were obtained with the rectified views to the original viewpoint space, and the matches before and after distortion rectification are fused to determine the matching pairs. In this way, the number of matching points can be greatly increased and the incorrect or repeated matches are discarded.

The rest of this paper is organized, as follows. The proposed matching method is described in detail in Section 2, while Section 3 presents the experimental results, the comparative evaluations, and a discussion. Finally, our conclusions are drawn in Section 4.

## 2. Materials and Methods

**Figure 2:**
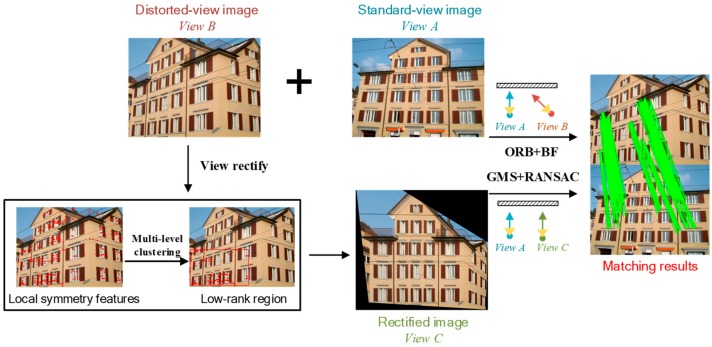
The workflow of the proposed method.

Figure 2 shows the workflow of the proposed method. Specifically, the main workflow is as follows: (1) firstly, the extracted local symmetry features, followed with multi-level clustering, are used to define the low-rank texture regions, and the distorted image is rectified with the TILT algorithm; (2) the ORB features are extracted from the images before and after rectification, and rough matches are obtained with the brute-force matcher; (3) the GMS and RANSAC techniques are then employed to discard the incorrect matches; and, (4) finally, the matching pairs before and after viewpoint rectification are fused to increase the number of effective matching pairs. 

### 2.1. Viewpoint Rectification with Automatic Low-Rank Texture Recovery

#### 2.1.1. Low-Rank Texture Recovery

The viewpoint rectification is implemented based on the theory of low-rank recovery. Generally speaking, a matrix $I^{0}$ defined on $R^{2}$ can represent a 2D image of size m×n. If the rank of $I^{0}$ satisfies the conditions given in Equation (1), where the rank $r\left(I^{0}\right)$ is much less than the dimensions of the matrix, then we can consider $I^{0}$ to be a low-rank texture.

(1) $$r\left(I^{0}\right)=rank\left(I^{0}\right)\ll \min\left(m,n\right)$$

Typically, the images of regular symmetric patterns confirm to the properties of low-rank textures. For the images containing urban building facades, they are mainly composed of regular shapes, symmetrical structures, and repeated textures (e.g., walls, windows, and fences) [41]. In the ideal case, these regular and repeated patterns can be extracted and represented by low-rank matrices [42]. However, real-world images usually suffer from deformation and corruption that are caused by the varying viewing angles, which deforms the symmetry and regularity of the image textures. Thus, the low-rank property of the symmetrical textures is also deformed. The TILT algorithm the Zhang et al. proposed [35] is an algorithm that is able to recover the low-rank textures in 2D images, despite significant corruption and warping through convex optimization of matrix rank minimization. The problem can be described, as follows:

(2) $$\underset{I^{0},E,\tau }{min}\;\mathrm{rank}\left(I^{0}\right)+\gamma {\left|\left|E\right|\right|}_{0}\;s.t.\;I\circ \tau =I^{0}+E$$

where I is the deformed and corrupted image and $I^{0}$ is the desired low-rank texture. The aim of the optimization function is to find the $I^{0}$ with minimized rank, with the known I being corrupted by the sparse error component E (i.e., noise, occlusions, and other non-Gaussian errors), and transformed with the projective transformation $\tau \;\left(R^{2}\to R^{2}\right)$. In the equation, ${\left|\left|E\right|\right|}_{0}$ is the $l_{0}$-norm, referring to the zero entries of E, and the parameter γ is a positive weight to balance the rank of the texture and the sparsity of the error. TILT exploits the best local deformation by minimizing the rank of the local brightness by solving the optimization problem in Equation (2), thus recovering the desired low-rank texture region $I^{0}$. However, the initialization of $I^{0}$ is crucial for desirable results. As noted in the introduction, the TILT algorithm requires a user-specified window for identifying the candidate low-rank regions in an image [35]. This is both time- and labor-consuming in real applications. Thus, the automatic extraction of low-rank texture regions is essential.

#### 2.1.2. Automatic Low-Rank Texture Extraction

Two principles need to be considered in low-rank texture extraction for urban building images. Firstly, the size of the low-rank region should be neither too large nor too small. There might be too much background information and noise involved, if the extracted low-rank region is too large, which would prevent us from obtaining an accurate transformation matrix. In contrast, the limited information contained in the local area might not be enough to calculate a transformation matrix representing the whole image if the extracted low-rank texture region is too small. Secondly, the low-rank texture should be limited to a single plane in the real 3D scene. In the image scenes containing the corners of the building (two sides of walls) or walls connecting with the ground, the extracted low-rank region might be cross-plane, which would thus lead to the failure of TILT [35]. 

We propose an efficient automatic low-rank texture extraction algorithm for urban building images based on these facts. The proposed algorithm includes the detection of local symmetry features and low-rank texture extraction that is based on multi-level clustering of the detected features.

A. Local Symmetry Feature Extraction

Local features refer to the image patterns that are associated with changes of image properties, which differ from their immediate neighborhood. The following requirements should be satisfied to extract the low-rank texture regions using the local feature points in the urban building images: (1) the detected feature points are mainly distributed on the building facades, rather than the other objects in the scene, e.g. trees, cars, or the side walls, to avoid unnecessary distractions; and, (2) the distribution of the feature points conforms to the symmetry and regularity of the building textures. Thus, we introduce the local symmetry features, which are a fundamental characteristic of building images, for the extraction of low-rank texture regions. The local symmetry features are based on measures of local bilateral and rotational symmetries [37]. The core idea is to detect a dense feature set through computing the local symmetry scores while using the local image operations over the image and across different scale spaces. The local symmetries are scored based on the local symmetry score function, which is defined as:

(3) $$SS\left(p\right)=L*\underset{q}{\sum }w_{\sigma }\left(\left\|q-p\right\|\right)\cdot d\left(q,M_{t,p}\left(q\right)\right)$$

where p=(x,y) is the point of interest and q=(x,y) varies over all of the pixel locations in the image with valid reflected pairs. The function $M_{t,p}\left(q\right)$ maps image point q onto its symmetrically corresponding point with respect to point p in the image. Here, t is the symmetry type, which, in this paper, mainly refers to horizontal- and vertical-bilateral symmetries. Correspondingly, L is the filtering kernel that is used to convolve the local symmetry distance (SD) map. Following the guidance in [37], the kernel is designed to use a Laplacian-of-Gaussian (LoG) profile [43] that is perpendicular to the symmetrical axis, and a Gaussian profile along the axis of symmetry. The gradient orientation histogram is used to evaluate the differences between the two reflected pixel positions q and $M_{t,p}\left(q\right)$. The comparison between histograms is computed with their dot product:

(4) $$d\left(q,M_{t,p}(q)\right)=h\left(q\right)\cdot h\left(M_{t,p}(q)\right)$$

where h(q) is the normalized vector-valued gradient orientation histogram function at point q. The larger $d\left(q,M_{t,p}\left(q\right)\right)$ is, the more similar the two histograms are. Despite the histogram function, a weight mask $w_{\sigma }$ is employed to confer more importance to the points closer to the point of interest, which effectively localizes features in the scale-space. The weight is usually defined as radially symmetric:

(5) $$w_{\sigma }(r)=Ae^{-\frac{r^{2}}{2\sigma^{2}}}$$

where r is the distance to p and A is a normalization constant. σ is the scale for the weight mask determining the size of its support region.

**Figure 3:**
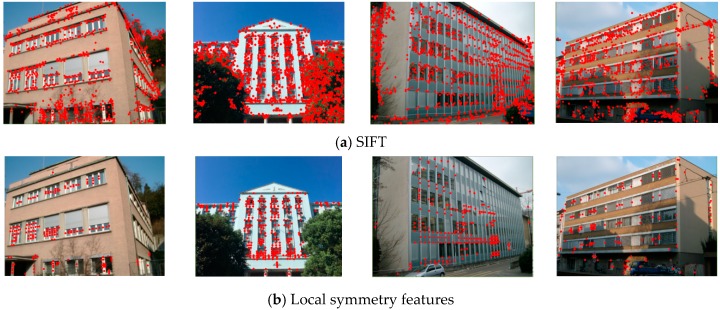
The difference between detected SIFT and local symmetry features.

The features are then detected based on the symmetry scores computed on a Gaussian image pyramid densely that was sampled in the scale-space. Firstly, the initially detected feature set F=(x,y,s) is obtained by discarding the points with small score values in each scale. Here, (x,y) denotes the pixel positions, while s is the scale, also referring to the radius of the support region in the original image. The features are then updated with locally strong detections across the preserved scales. This is implemented by keeping each detected feature $F_{i}$, if it has the highest symmetry score among the features whose support region overlap that of $F_{i}$ by more than 0.4. The support region here is defined as the circle of radius s that is centered at $\left(x_{i},y_{i}\right).$ We give some examples of the final detection results that were obtained while using the local symmetry features and SIFT detector in Figure 3. It can be observed from the results that the local symmetry features are capable of excluding most of the unnecessary detected feature points on the side walls, trees, and sky.

B. Automatic Low-Rank Region Detection Using Multi-Level Clustering

Although the local symmetry features show a robust performance in feature detection for the urban building scenes, there are still outliers that are extracted on the non-target objects. As shown in Figure 3, some feature points are distributed on the side walls and the car near the building facade. These outliers would result in oversized low-rank texture regions being extracted, thus affecting the accuracy of the view rectification matrix. In this section, we propose using a multi-level clustering method for the detected local symmetry features, thus removing the undesired outliers and retaining the effective features for the low-rank texture.

(1) Mean Shift Clustering

The multi-level clustering is implemented based on the mean shift technique [44], which is a popular non-parametric clustering technique for feature space analysis that does not require us to predefine the number of cluster centers. The core idea of the mean shift technique is assuming that the data points in the feature space are sampled from an underlying probability density function (PDF) and, thus, a high data density indicates the modes (local maxima) of the underlying distribution. For each data point, the mode detection is implemented with an adaptive gradient ascent procedure, and the modes of the density can be located with the convergence of the iterative procedure.

A kernel density gradient estimation method is used for the density estimation. Given n data points $x_{i}$ in the d (dimensional space), the kernel point estimator can be expressed as:

(6) $$f_{h,K}\left(x\right)=\frac{c_{k,d}}{nh^{d}}\sum_{i=1}^{n}k\left({\left\|\frac{x-x_{i}}{h}\right\|}^{2}\right)$$

where k(x) is a monotonically decreasing function, also referring to the profile of the kernel K. Moreover, h is the bandwidth value and $c_{k,d}$ is the positive normalization constant, which ensures that K(x) is integrated to one. The density gradient estimator is then obtained by exploiting the linearity of $f_{h,K}\left(x\right)$. When the derivative of the kernel profile k(x) exists, the function $g\left(x\right)=-k^{\prime }\left(x\right)$ can be defined. With the profile g(x), the kernel is defined as $G\left(x\right)=c_{g,d}g\left({\left\|x\right\|}^{2}\right)$, where $c_{g,d}$ is the corresponding normalization constant. The mean shift vector is then calculated by taking the gradient of the kernel estimator:

(7) $$m_{G}\left(x\right)=\frac{\sum_{i=1}^{n}x_{i}g\left({\left\|\frac{x-x_{i}}{h}\right\|}^{2}\right)}{\sum_{i=1}^{n}g\left({\left\|\frac{x-x_{i}}{h}\right\|}^{2}\right)}-x$$

where x is the center of the kernel and $x_{i}$ denotes the other points within the kernel. The mean shift vector is the difference between the weighted mean and the center point, thus the local mean are shifted toward the direction of maximum density [44,45]. The mean shift procedure is applied to each data point in the feature space. When a new cluster center $C_{new}$ is detected, the distances between $C_{new}$ and the existing centers are calculated. If the distance between $C_{new}$ and $C_{i}$ is smaller than the threshold d, the two clusters are then merged; otherwise, the new cluster for $C_{new}$ is preserved.

(2) Multi-Level Clustering Based on Mean Shift

**Figure 4:**
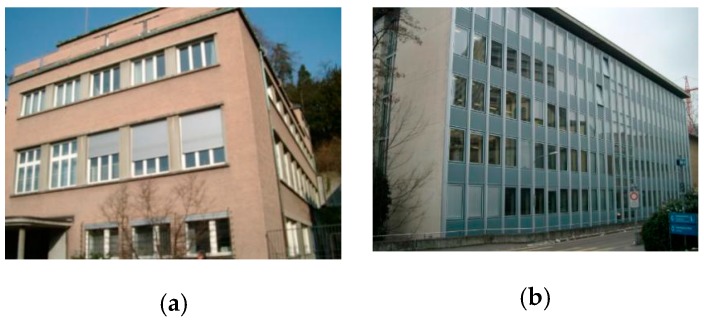
Building facades with different textures. (**a**) A building wall with dividing units between the windows, and (**b**) a building wall with no distinct dividing units between the windows.

The multi-level clustering algorithm that is based on mean shift is designed for the refined selection of the detected local symmetry feature points. The detailed steps are as follows:

(i) Use mean shift to cluster the initial detected local symmetry feature points, and sort the clusters $C=\left\{C_{1},C_{2},C_{3},\cdots \right\}$ by the feature density.
(ii) Select the feature cluster with the highest density $C_{1}$ as the primary cluster for the extraction of the low-rank texture regions, and the others are considered to be the candidates. The textural characteristics of the building facades determine the number of candidate clusters. In this paper, we classify the texture of the building facades into two categories, depending on whether there are dividing units between the windows, as shown in Figure 4. For the case where spaces exist between the windows (Figure 4a), $C_{2}$ is selected as the candidate cluster, which is because the dividing space would prevent the shift of the cluster centers. It is unnecessary to use candidate units for the latter (Figure 4b) case with no evident dividing units between the windows.
(iii) For the selected feature points in the first-level clustering, there might be remaining discrete noise points. Thus, a mean shift is applied again to the obtained primary and subordinate clusters, respectively. In this step, threshold δ≥10 is set to constrain the second-level clustering procedure. As a result, only the top-two dense clusters with more than 10 feature points are counted, referring to $C_{i,j}$, where i and j represent the serial number of the clusters in the first- and second-level clustering procedures.
(iv) Furthermore, the candidate feature points are constrained with the bounding range of the primary cluster for the building facades with a non-rectangular 2D geometric shape, avoiding the candidate clusters of feature points located outside the range of the central low-rank texture region.

The proposed method uses local symmetry features and multi-level clustering to automatically locate the low-rank texture regions, and it thus provides an effective solution to the efficiency and practicability of TILT. The proposed strategy extracts local feature points, instead of the geometry of the building facades, which is more simple and efficient when compared with the currently popular methods using multi-feature fusion, facade segmentation, and repeated pattern detection.

### 2.2. Image Matching and Viewpoint Fusion

#### 2.2.1. Image Matching

Image matching can then be implemented with the two images with rectified views after image rectification. Generally speaking, local feature based matching consists of three main steps: (1) feature extraction from individual images; (2) feature matching for image pairs; and, (3) outlier removal for match refinement. Among the numerous image matching methods, the most commonly employed approach is based on SIFT feature detection and matching, followed by the removal of wrong matches (outliers) with the RANSAC technique [40,46]. These technological processes can achieve good results in the usual cases, where no distinctive distortion exists between the images. However, for the images with large projective distortions, the situation is more complex. Although the viewpoint differences are eliminated with the image rectification, information differences still exist. This results in significant differences between the descriptors for the corresponding feature points, thus increasing their distance. In such a case, image matching while using the SIFT and RANSAC techniques might fail, due to the insufficient number of feature points detected and the excessive wrong matches disturbing the iterative parameter estimation process for the RANSAC algorithm. 

In the proposed method, based on all of these facts, image matching is implemented while using the rich and cost-effective ORB features, and the GMS and RANSAC techniques for the purification of the rough matching results [38,39]. The ORB feature descriptor is a local binary feature descriptor that was developed based on the “features from accelerated segment test” (FAST) keypoint detector [47] and the “binary robust independent elementary features” (BRIEF) descriptor [48]. The detected ORB features are roughly matched while using the brute-force matcher. The GMS technique is then used to roughly reject the wrong matches, as well as to prevent the discarding of correct matches. In this way, the proportion of correct matches is increased. Generally speaking, the true/false matches can be distinguished by the number of matches in their neighborhood. The core idea of the GMS technique is to incorporate the motion smoothness constraints (neighboring pixels share similar motion for the images with different viewpoints) into a statistical framework to reject the wrong matches [39]. The motion smoothness assumption implies that the neighborhoods of a true match view the same 3D region. Given an image pair $\left\{I_{a},I_{b}\right\}$ with {N,M} features, respectively, $Q=\left\{q_{1},q_{2},\ldots ,q_{N}\right\}$ is the set of feature matches from $I_{a}$ to $I_{b}$, and $Q_{i}\subseteq Q$ represents the subset of matches between regions {a,b} of match $q_{i}$, where a and b are the corresponding supporting regions of match $q_{i}$ in the images. We denote $f_{a}$ as one of the n supporting features in region a, with a probability of t to be a correct match. If $f_{a}$ matches wrongly ($f_{a}^{f}$), its correct match can lie in any of the M locations in image $I_{b}$. Thus, the probability for its nearest-neighbor match lying in region b ($f_{a}^{b}$) can be expressed as:

(8) $$p\left(f_{a}^{b}|f_{a}^{f}\right)=\beta m/M$$

where m is the number of features in region b and β is the constant to accommodate violations of the above assumption caused by repeated structures. If event $T^{ab}$ denoted as {a,b} views the same 3D location, then:

(9) $$p_{t}=p\left(f_{a}^{b}|T^{ab}\right)=t+\left(1-t\right)\beta m/M$$

Likewise, $F^{ab}$ is defined to be the event of {a,b} viewing a different 3D location, and:

(10) $$p_{f}=p\left(f_{a}^{b}|F^{ab}\right)=\beta \left(1-t\right)\left(m/M\right)$$

Given that motion is usually smooth over a large region rather than a small neighborhood, then a true match allows for multiple small region pairs to view the same 3D location. Correspondingly, using the sample prediction function on a false match would cause geometrically different locations. The generalized measurement score of the neighborhood support can be computed as:

(11) $$S_{i}=\sum_{k=1}^{K}\left|Q_{a^{k}b^{k}}\right|-1$$

where K is the number of disjoint regions that $q_{i}$ predicts move together. The predicted region pairs are expressed with $\left\{a^{k},b^{k}\right\}$, while $Q_{a^{k}b^{k}}$ is the subset of matches within region pair $\left\{a^{k},b^{k}\right\}$.

Following the idea of the GMS technique, we divide the rectified image to be matched into G×G grids, which was set to 20 in this paper. The score $S_{i,j}$ for cell pair {i,j} is:

(12) $$S_{i,j}=\sum_{k=1}^{K=9}\left|Q_{i^{k}j^{k}}\right|$$

where the number of neighborhood regions around cells {i,j} is set to 9 (3×3 grid) and $\left|Q_{i^{k}j^{k}}\right|$ is the number of matches between cells $\left\{i^{k},j^{k}\right\}$. Finally, a threshold is applied to $S_{i,j}$, thus dividing the cell pairs into true and false matching sets {T,F}:

(13) $$cell-pair\left\{i,j\right\}\in \left\{ \begin{array}{ll} T, & {\mathrm{if}\;S}_{ij}>\tau_{i}=\alpha \sqrt{n_{i}}\\ F, & \mathrm{otherwise} \end{array}\right.$$

where $n_{i}$ is the number of features in the grid cell. Empirically, the coefficient α is set as large enough to ensure that most of the wrong grid cells are removed. In this paper, we empirically set α as six. 

#### 2.2.2. Viewpoint Fusion for Image Dense Matching

**Figure 5:**
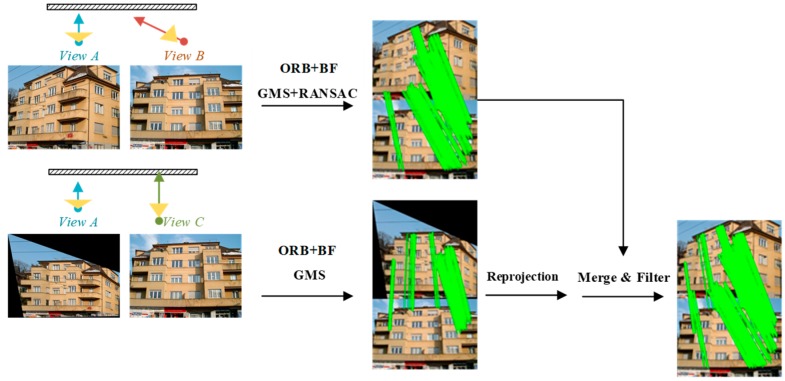
Viewpoint fusion for image dense matching.

We propose a viewpoint fusion strategy to increase the number of matching pairs to further improve the image matching quality. The image matching procedure is conducted in both the original and the rectified views. For the original image pairs, GMS may produce incorrect motion statistics for the repeated texture regions, due to the large changes in viewpoint, thus leading to inaccurate matching results. In this case, RANSAC is applied to further refine the matching result. The matching pairs for the rectified images are then normalized to the original view space, and the matching pairs that were obtained in different viewpoints are fused, as shown in Figure 5. We set $Q_{1}=\left\{q_{1}^{1},q_{2}^{1},\cdots ,q_{n}^{1}\right\}$ and $Q_{2}=\left\{q_{1}^{2},q_{2}^{2},\cdots ,q_{m}^{2}\right\}$ as the set of feature matches for the original and rectified images, respectively. n and m indicate the number of feature matches for the corresponding image pairs. $q_{i}^{j}=\left(p_{i}^{k},p_{i}^{A}\right)$(Here, $p_{i}^{k}={\left[x,y,1\right]}^{T}$ is the homogeneous coordinates of the matching point) denotes the feature match, where i is the index number for the matching points, and j∈{1,2} is the index for the matching sets. k∈{B,C} is the index for the viewpoint, where A, B, and C indicate the orthographic view, the distorted view, and the rectified view, respectively. 

Firstly, the matching point $p_{i}^{C}$ is projected to the distorted view B with the transform-obtained matrix H (see Section 2.1.1), i.e., ${\left(p_{i}^{B}\right)}^{\prime }=H\times p_{i}^{C}$. Thus, the point set can be converted to ${\left(Q_{2}\right)}^{\prime }=\left\{\left({\left(p_{1}^{B}\right)}^{\prime },p_{1}^{A}\right),\left({\left(p_{2}^{B}\right)}^{\prime },p_{2}^{A}\right),\cdots ,\left({\left(p_{m}^{B}\right)}^{\prime },p_{m}^{A}\right)\right\}$. We traverse ${\left(Q_{2}\right)}^{\prime }$ for each matching pair $\left({\left(p_{i}^{B}\right)}^{\prime },p_{i}^{A}\right)$ and search for the nearest match in set $Q_{1}$. If the searched matching points satisfy the distance threshold (five pixels in this paper), they are deleted. In this way, the repeat matching pairs can be deduced, and the final matching results are obtained by merging the processed sets $Q_{1}$ and ${\left(Q_{2}\right)}^{\prime }$.

## 3. Experiment Results

In this section, the experimental results and analysis are presented to validate the effectiveness of the proposed method. In this study, the Zurich building dataset (ZuBuD) [36] and a local dataset are utilized in our experiments. The experiments were mainly composed of two parts to illustrate the contribution of each step in the proposed method: (1) the automatic low-rank texture region extraction algorithm was first tested, and the effectiveness of the local symmetry features and multi-level clustering was evaluated; and, (2) the improvements in image matching performance with viewpoint rectification and fusion were then validated on the images with large perspective distortions. The representative feature matching methods were included for comparison. 

### 3.1. Data Preparation and Implementation

**Figure 6:**
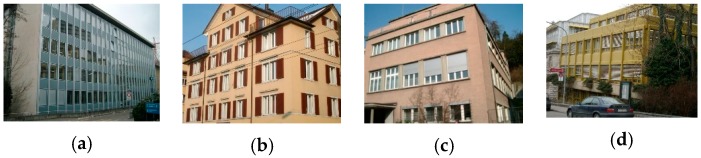
Example images with discriminative wall textures from the ZuBuD dataset. (**a**) image pair 1; (**b**) image pair 2; (**c**) image pair 3; (**d**) image pair 4.

**Figure 7:**
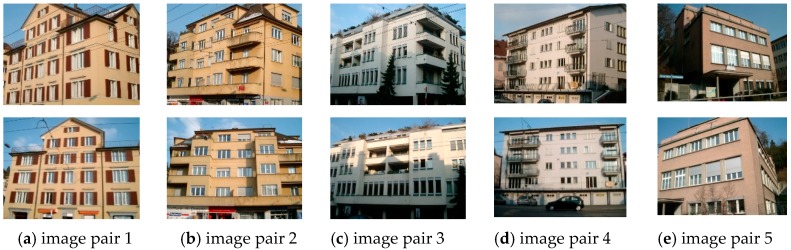
Five image pairs with large view differences from the ZuBuD dataset.

**Figure 8:**
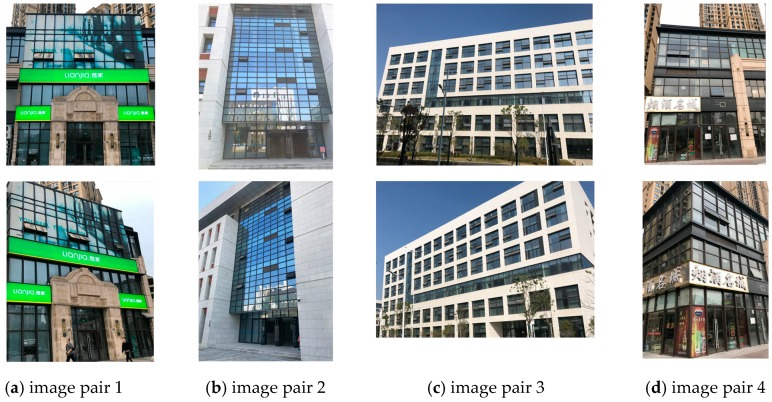
Four image pairs with different image size and different architecture style of buildings from the local dataset.

The ZuBuD dataset consists of 1005 images (640 × 480) for 201 buildings in Zurich, where each building image was captured from five different views with varying weather and illumination conditions. We chose four pairs of images while considering the data characteristics, where the building images have very different wall textures and are occluded by different objects (e.g., cars and trees, as shown in Figure 6), for the low-rank texture region extraction and viewpoint rectification. In the matching experiments, five image pairs with significant viewpoint differences were selected for validating the effectiveness of the proposed method in complex cases, as shown in Figure 7. In addition to the ZuBuD dataset, we also collected building images from local environments for experiment, as shown in Figure 8. These images are also captured from buildings with different viewpoints, occlusions, and illumination conditions. 

All of the experiments were conducted on a personal computer that was configured with an Intel(R) Core (TM) i7-7820X 3.60 GHz CPU, 8 GB RAM, and 64-bit Microsoft Windows 10 operating system. The program was written with the MatLab code, in cooperation with the OpenCV 3.4 library. Table 1 provides the main parameters used in our experiment. 

### 3.2. Results on ZuBud Dataset

#### 3.2.1. Image Rectification

The four images shown in Figure 6 with discriminative wall textures and occlusions were first selected in the experiments in order to assess the effectiveness of the proposed low-rank texture region extraction algorithm and the viewpoint rectification. There are no clear dividing units between the windows in the building walls in Figure 6a, and the lateral wall is a homogeneous region with no distinctive textures. Comparatively, the windows in the building facade of Figure 6b are segmented by the wall in yellow, and the 2D geometric shape of the building is non-rectangular. Moreover, it can be observed that the buildings in Figure 6c,d are occluded by other non-target objects, including telegraph poles, cars, and branches.

**Figure 9:**
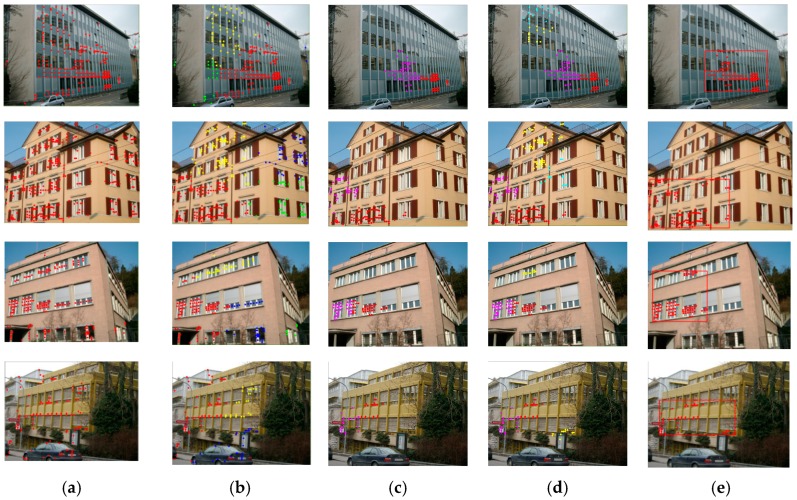
Low-rank region extraction results based on local symmetry feature points and multi-level clustering. (**a**) The original detected local symmetry feature points. (**b**) The initial clustering results obtained using mean shift, where the red and yellow points indicate the primary and candidate classes. (**c**,**d**) The second-level clustering results of the feature points for the primary and candidate classes. The red and magenta points represent C_11_ and C_12_, and the yellow and green points in (**d**) indicate C_21_ and C_22_, respectively. (**e**) The final determined low-rank texture region.

For the representative images, Figure 9 shows the feature detection and low-rank texture region extraction results that were obtained using the proposed algorithm. With the initial feature points (the red points in Figure 9a), the results for the multi-level feature clustering and filtering processes are provided in Figure 9b–d. In the first-level clustering, the initial detected features are clustered based on distribution density while using mean shift, where the classes with density from high to low are displayed with red, yellow, blue, and green points, respectively. The cluster with the highest point density is selected as the primary class, while the second-highest one is used as the candidate class. The primary and candidate classes are then separately clustered and filtered, which is denoted as the second-level clustering. Generally speaking, the top-two classes in point density (the red and magenta points in Figure 9c and the yellow and green points in Figure 9d) are selected and then merged to construct the final feature set (the red points in Figure 9e). It is worth mentioning that, for the first image with no dividing units between the windows, only the primary class of features (the red points in Figure 9b) is considered in the second-level clustering, as described in Section 2.1.2. With the detected local symmetry features, the low-rank texture region is determined with the bounding box of the point set. The spatial range of the primary class of feature points is used as the constraint for the selection of the candidate class of features for the buildings with a non-rectangular 2D geometric shape, as shown in the second row of Figure 9.

**Figure 10:**
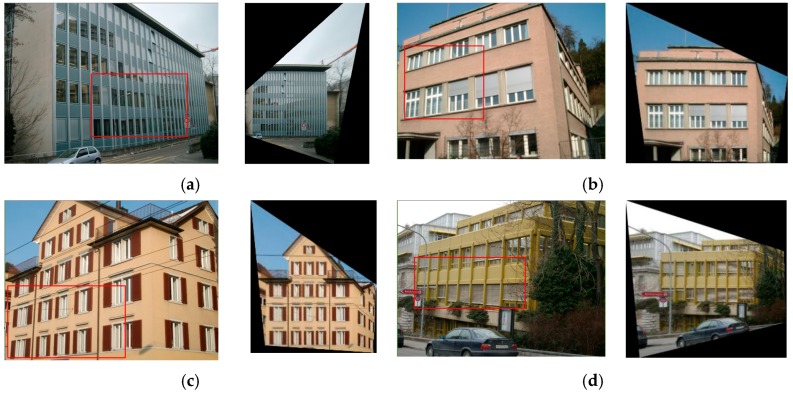
The results of image rectification. (**a**)–(**d**) are the four images before and after low-rank matrix recovery with the transform invariant low-rank textures (TILT) algorithm.

**Figure 11:**
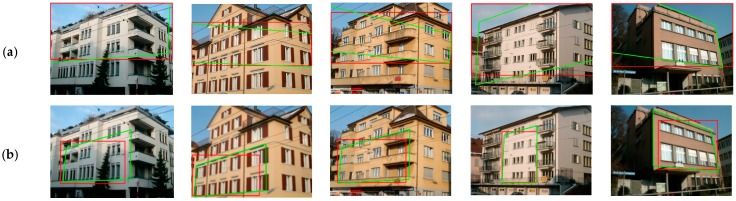
The extracted low-rank texture regions and the results processed by the TILT algorithm. (**a**) The results obtained with the method proposed in [34]. (**b**) The results obtained using the proposed low-rank texture region detection algorithm. The red lines denote the input to the TILT algorithm, and the green lines denote the returned deformed textures.

Based on the low-rank texture regions, the image view can be rectified while using the TILT algorithm. As shown in Figure 10, the left image shows the image with deformation due to the viewpoint, while the right one is the output with view rectification. The red rectangular regions are the low-rank regions. In Figure 11, we present a detailed analysis of the low-rank recovery algorithm. The green lines indicate the regions after rectification. The results obtained following the method that was proposed by Zhang et al. [34] are included for comparison. In their work, they proposed using parallel lines that were extracted by rotating images with multiple angles to determine the borders of the low-rank texture regions. However, simply using image rotation is not capable of simulating the deformation to image textures caused by perspective transformation. Moreover, the method might fail in the complicated cases, without consideration of the occlusions or the multiple wall planes in the image scenes. As a result, it can be seen that the detected low-rank regions that were obtained using Zhang’s method are generally too large, and the detected regions contain too many occlusions, or are cross-plane, and are thus unable to represent the intrinsic low-rank textures. In these cases, the inaccurate low-rank texture region input to the algorithm will lead to the failure of its convergence to the correct solution, as the initial low-rank texture regions are vital to the TILT algorithm. 

**Figure 12:**
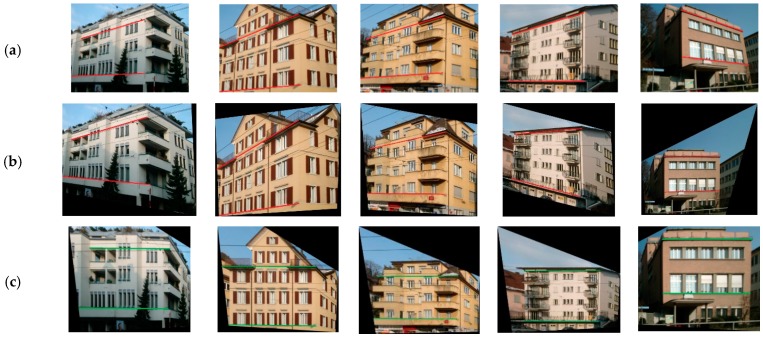
Comparative results of the image rectification. The lines marked in the images are the parallel lines in the real scenes. (**a**) Original; (**b**) Zhang et al. [34]; (**c**) Proposed.

We can obtain the transformation matrix and the whole image can then be rectified with the effective low-rank regions detected. We extracted the parallel lines on the building facades, as shown in Figure 12. It can be clearly observed that the lines are not parallel in the original image with projective distortions, as well as the rectified results that were obtained with Zhang’s method proposed in [34]. Comparatively, the results of our method can effectively recover the deformed building structures and textures, and thus the lines are parallel, as in real 3D scenes. In this way, the feature information differences that are caused by the influence of image distortion can be reduced.

#### 3.2.2. Matching Results

The image pairs that were used in these experiments had large perspective distortions (Figure 7), thus bringing extra difficulties to the matching. In Section 3.2, we showed that a distorted image can be effectively rectified to the standard view with the proposed automatic low-rank region detection method and the TILT algorithm. We conducted two groups of experiments to illustrate the efficacy of the proposed method, in terms of viewpoint rectification and fusion strategy, respectively. Firstly, the commonly used image matching methods of SIFT [15], speeded up robust features (SURF) [16], AKAZE [49], affine SIFT (ASIFT) [18], and binary robust invariant scalable keypoints (BRISK) [50] were tested on the original and rectified images, respectively. The statistics are used to quantitatively evaluate the results (Table 1), i.e., the total matches $N_{T}$, the correct matches $N_{C}$, the feature repeatability rate ($F_{R}$) of detected features, and the correct matching rate ($C_{R}$). It is noted that $N_{T}$ indicates the matches that are returned by the outlier removal techniques, and the correct matches are further selected by manual visual examination. $C_{R}$ is defined as the ratio between the number of correct matches and the total matches, and $F_{R}$ is denoted as the ratio between the number of correct matches and the minimum number of detected feature points from the two images to be matched, which can be expressed as:

(14) $$\begin{array}{l} C_{R}={N_{C}/N}_{T}\\ F_{R}=N_{C}/\min\left(N_{ref},N_{test}\right) \end{array}$$

where $N_{ref}$ and $N_{test}$ indicate the number of extracted feature points from the reference image and the image to be matched, respectively. From the results that are listed in Table 2, it can be seen that the traditional feature descriptors have difficulty in obtaining good results due to the information differences between the images that are caused by the large projective distortions. In fact, there are no correct matches in some cases. The feature repeatability rates are generally low due to the large viewpoint difference, which indicates significant information differences between the image scenes. 

**Figure 13:**
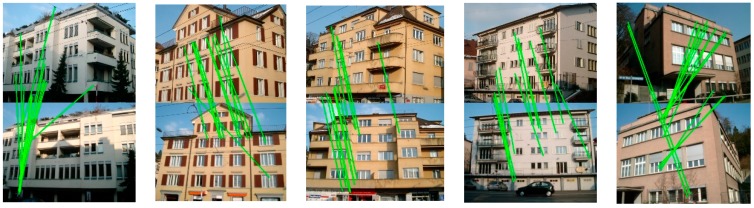

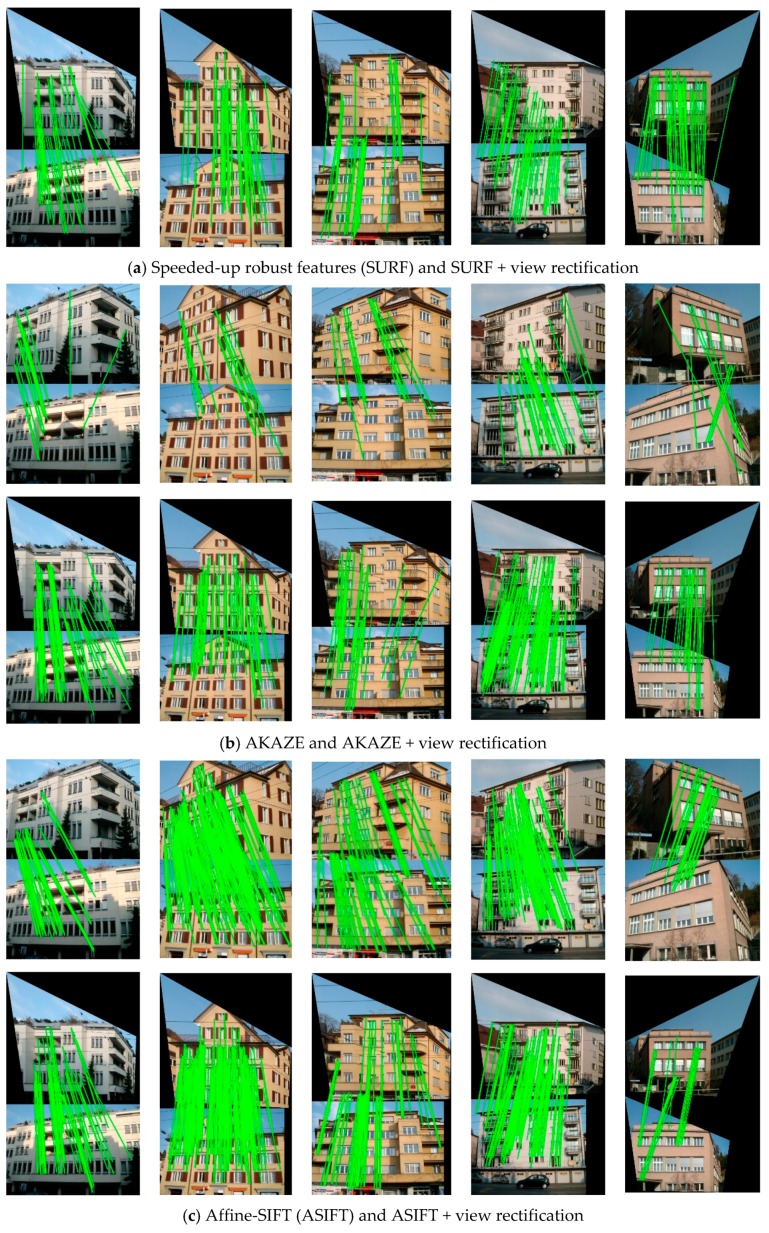
Improvement of the matching results with viewpoint rectification. (**a**)–(**c**) are the image matching results before and after viewpoint rectification for SURF, AKAZE and ASIFT, respectively.

We present the visualized matching results of SURF, AKAZE, and ASIFT in Figure 13. In terms of the feature descriptors’ performances, these three methods all obtain relatively good results for all of the test images. With view rectification while using the TILT algorithm, the number of total and correct matching points is significantly increased. In most cases, improvements can also be found regarding the correct matching rate $C_{R}$. From Figure 13, it can be observed that the deformed image textures can be effectively recovered after image rectification, and the matches are more uniformly distributed across the whole image. Among the feature detectors, ASIFT is superior to the others, showing a robust performance for the distorted image matching, with $C_{R}$ being above 90% in all the cases. From Table Improvements, the number of correct matches and the feature repeatability ratio ($F_{R}$) can also be observed.

**Figure 14:**
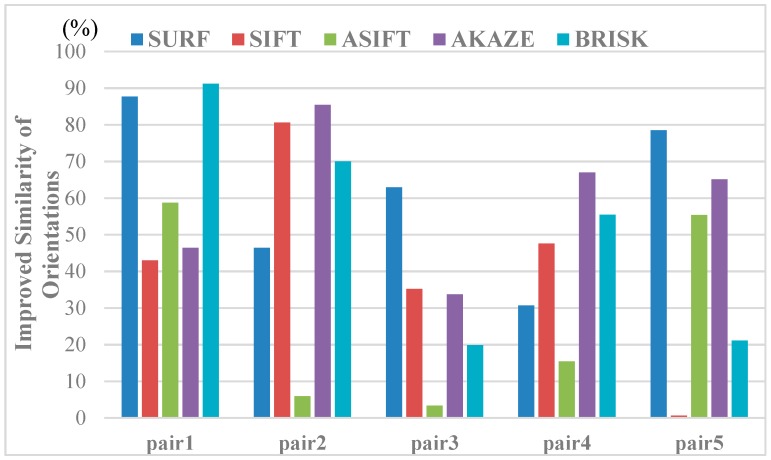
Results of improving the orientation similarity of all the kinds of feature matching by viewpoint rectification.

**Figure 15:**
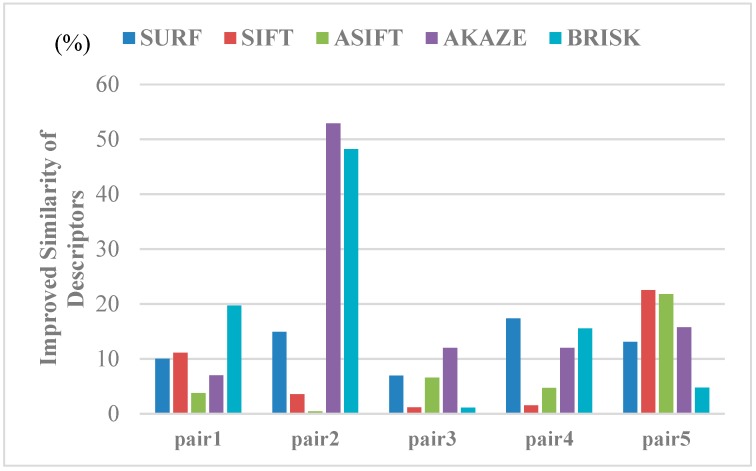
The results of improving the similarity of the descriptors by viewpoint rectification.

We quantitatively evaluated the similarity between the corresponding points in the light of the feature orientations and descriptor distances to further illustrate the effect of the image rectification for the improvement of the correct matching rate. The indices used can be calculated, as follows:

(15) $$\begin{array}{l} M\_ori=\frac{1}{n}\sum_{i=1}^{n}\left(ag_{i}^{1}-ag_{i}^{2}\right)\\ M\_des=\frac{1}{n}\sum_{i=1}^{n}\left({\left\|des_{i}^{1}-des_{i}^{2}\right\|}_{2}\right) \end{array}$$

where M_ori and M_des denote the mean differences between the correspondences for the matching image pairs and n is the total number of matching pairs. Among them, $ag_{i}^{k}$(k=1,2) is the feature orientation of the ith keypoint belonging to the set of corresponding points in the image pairs. Similarly, $des_{i}^{k}$ indicates the vector form of the feature descriptor for the ith corresponding point. The mean differences were then calculated with the Euclidean distance of the descriptor vectors. The mean differences for the image pairs before and after image rectification were obtained to evaluate the influence of the view rectification on the image matching, as in Equation (15), and we give the improvement rates of the similarity between the corresponding features in Figure 14 and Figure 15. From the results, both of the indices are improved, to varying degrees, and significant improvements can be observed in the similarity of the feature orientations. For the similarity of the feature orientations, the improvement rate is over 20% in most cases. The similarity of the feature orientations after image rectification shows a sharp increase of over 90% for the BRISK algorithm. This indicates that, with the viewpoint rectified, the perspective distortion of the textures in the original image has been corrected effectively.

**Figure 16:**
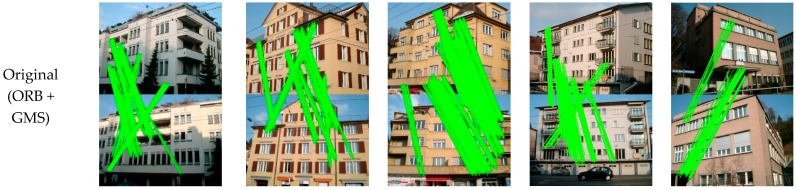

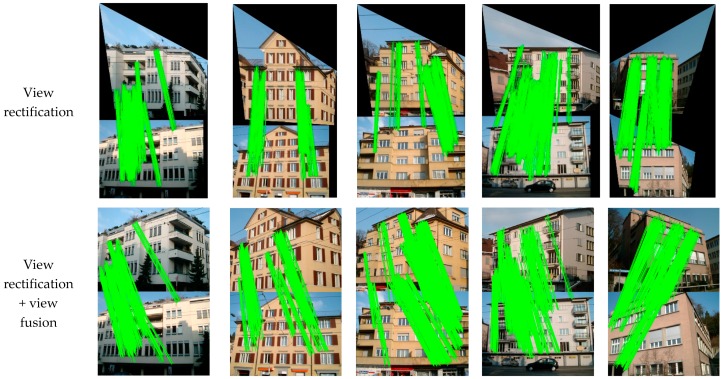
Comparative results of the feature point matching.

The overall performance of the proposed method was analyzed in the second group of experiments. We present the visualized matching results with the different processing methods in Figure 16, and the quantitative results are listed in Table 3, to illustrate the necessity of viewpoint fusion. In the first row of Figure 16, we can see that there are a lot of wrong matches. Although the GMS technique has been employed, the large perspective deformations between the image pairs significantly decrease the correct matching rate for most scenarios. With the viewpoint rectified, the matching performance shows a significant improvement in most cases. However, the distortions in the real image scenes are complex. For example, for image pair 3, the matching for the rectified views does not obtain good results, which is possibly due to the unsatisfactory view angle of the standard image. With the fusion of the information in different views, the viewpoint fusion can further increase the number of matching pairs, and it ensures that the method is robust. The superiority of the proposed method is obvious, in terms of both the numbers of total matches and correct matches, when compared with the commonly used feature detectors (Table 2 and Figure 13). For image pair 3 after image rectification, the ASIFT algorithm obtains 82 correct matches, while the proposed method obtains 1075 correct matches. The improvement is also prominent when compared with the results without view rectification and fusion (752 correct matches for image pair 3). In addition, the correct matching rate and the feature repeatability rate are generally high for the results that were obtained by the proposed method, which further verifies the effectiveness of the proposed matching method.

### 3.3. Results on Local Dataset

**Figure 17:**
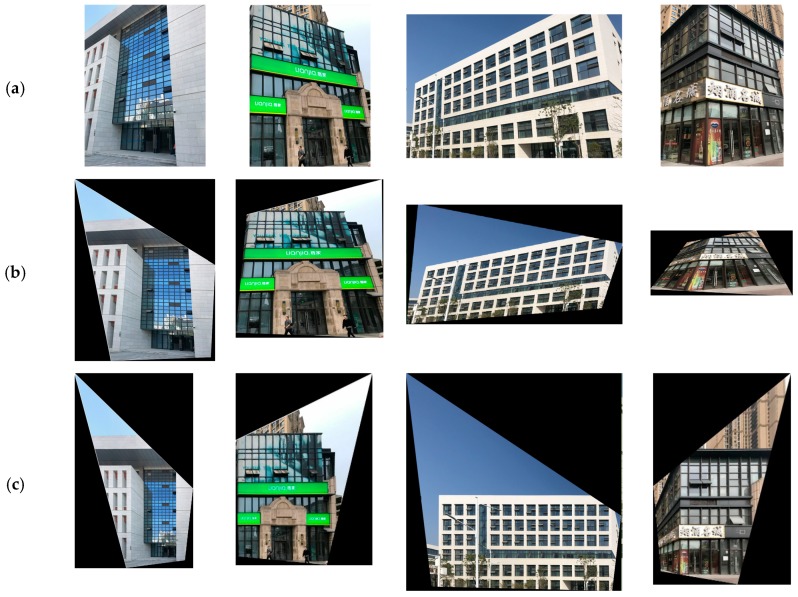
Comparative results of the image rectification on the local dataset. (**a**) Original; (**b**) Zhang et al. [34]; (**c**) Proposed.

The experiments were also conducted on the local dataset that is shown in Figure 8. The buildings from the local dataset have large differences in architectural styles with the buildings from the ZuBuD dataset, and can thus further validate the effectiveness of the proposed method. Figure 17 shows the results of image rectification with the proposed automatic low-rank texture extraction and TILT algorithm. Zhang’s method proposed in [34] was also chosen for comparison. From Figure 17, it can be seen that the Zhang’s method failed in most of cases, especially for the image shown in the last column. Whereas, all of the images are successfully rectified by using the proposed method, as shown in the third row of Figure 17. It demonstrated that the proposed automatic low-rank texture extraction can work well on building images with different architecture style and image size. 

**Figure 18:**
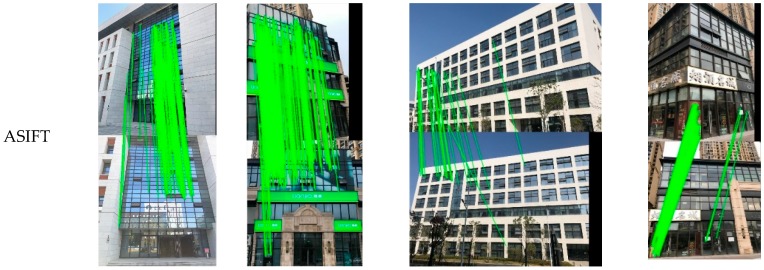

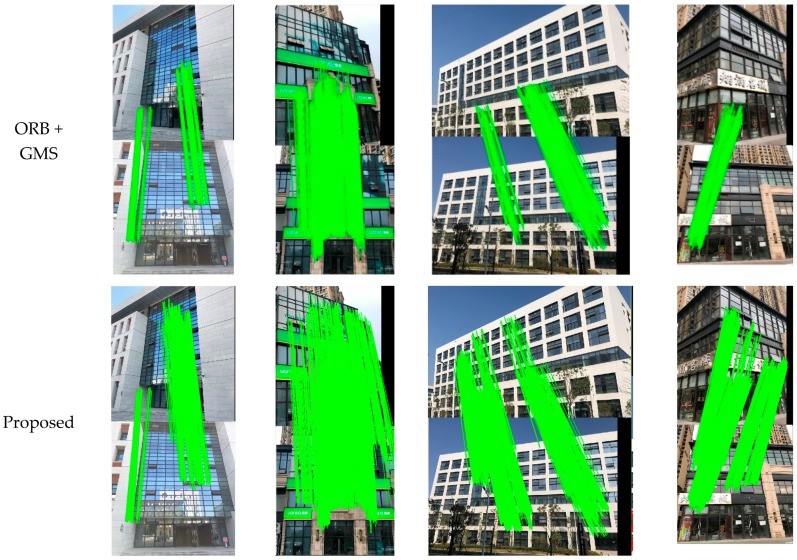
Matching results of the ASIFT, grid-based motion statistics (GMS) and the proposed method on the local dataset.

Figure 18 shows the final matching results. Given that the ASIFT and GMS method have better performance in all of the tests in Section 3.2, only the two methods were chosen for comparison in this section. From Figure 18, it can be seen that the performance of ASIFT is not very stable, especially for image pair 3 (fourth column), where the obtained matches are very sparse. For the original GMS, the matches are prone to aggregation in a small local region, especially for image pair 4 (last column). When compared to the above two methods, the proposed method has a robust performance, which can obtain a denser matching with the well-distributed matches. 

Table 4 provides a comparative analysis on the number of matches and the computation time for the three methods as a reference. The computational efficiency of the proposed method is higher than ASIFT (implemented in C++), but it is is relatively lower than GMS (implemented in Python). It is to be mentioned that the current implementation of the proposed method was written with MatLab code without any acceleration techniques, it still has a strong potential to improve the efficiency by a C++ implementation with GPUs. While considering the effective number of matches, as well as the cost time, the proposed method is practical. 

## 4. Conclusions

In this paper, we have proposed a wide-baseline image matching method that is based on viewpoint rectification and fusion. We propose to use local symmetry features, followed with multi-level clustering to automatically detect the low-rank texture regions to adapt the TILT algorithm for the image rectification of real building images with complex wall textures and structures. After image view rectification with the TILT algorithm, the brute-force matcher is employed to match the feature points that were detected with the ORB descriptor in the process of matching. The GMS and RANSAC techniques are then used to remove the outliers, as well as to prevent discarding of correct matches. Moreover, the matching results that were obtained with the rectified views are projected to the original viewpoint space, and the matches before and after distortion rectification are fused to ensure a sufficient number of matches. The proposed method is a practical way to match the urban building images with large projective distortions. The experimental results confirmed that the proposed method shows a robust performance in scenes with complex wall textures and occlusions.

## Figures and Tables

**Table 1:** The values of the main parameters used in the experiment.

	Metrics	Multi-Level Clustering Threshold	Maximum Number of ORB Features	Number of GMS Grids	Rejection Distance for Viewpoint Fusion
Parameters					
Value		10	100,000	20 × 20	5 pixel

**Table 2:** The quantitative evaluation results for the image matching with the classical feature descriptors before and after viewpoint rectification.

Test Images	Metric	SIFT		SURF		AKAZE		BRISK		ASIFT	
		Origin	Rectif	Origin	Rectif	Origin	Rectif	Origin	Rectif	Origin	Rectif
Pair 1	$N_{c}/N_{T}/Min\left(N_{L},N_{R}\right)$	0/12/1102	5/16/1102	0/14/1937	40/43/1937	18/20/1090	37/58/1090	13/19/1430	35/35/1430	64/65/20694	91/93/20694
	$C_{R}$	0.00%	31.25%	0.00%	93.02%	90%	63.79%	68.42%	100%	98.46%	97.85%
	$F_{R}$	0.00%	0.45%	0.05%	2.07%	1.65%	3.39%	0.91%	2.45%	0.31%	0.44%
Pair 2	$N_{c}/N_{T}/Min\left(N_{L},N_{R}\right)$	22/22/1416	41/44/1416	9/12/2210	47/48/2210	14/14/1150	56/56/1243	17/18/1466	38/38/1466	256/256/25545	264/264/25545
	$C_{R}$	100%	93.18%	75%	97.92%	100%	100%	94.44%	100%	100%	100%
	$F_{R}$	1.55%	2.90%	0.41%	2.13%	1.22%	4.51%	1.16%	2.59%	1.00%	1.03%
Pair 3	$N_{c}/N_{T}/Min\left(N_{L},N_{R}\right)$	16/17/1607	22/23/1607	15/16/2355	28/28/2355	14/15/1055	27/33/1126	19/21/1585	38/39/1585	77/77/29824	82/83/29824
	$C_{R}$	94.12%	95.65%	93.75%	100%	93.33%	81.82%	90.48%	97.44%	100%	98.80%
	$F_{R}$	1.00%	1.37%	0.64%	1.19%	1.33%	2.40%	1.20%	2.40%	0.26%	0.28%
Pair 4	$N_{c}/N_{T}/Min\left(N_{L},N_{R}\right)$	5/12/1799	53/53/1799	10/12/2168	49/49/2168	24/30/1358	108/108/1471	13/16/1902	109/109/1902	140/140/28142	148/148/28142
	$C_{R}$	41.67%	100%	83.33%	100%	80%	100%	81.25%	100%	100%	100%
	$F_{R}$	0.28%	2.94%	0.46%	2.26%	1.77%	7.34%	0.68%	5.73%	0.50%	0.53%
Pair 5	$N_{c}/N_{T}/Min\left(N_{L},N_{R}\right)$	0/12/1081	6/14/979	2/14/2195	41/43/1733	1/10/792	24/25/893	0/14/965	0/22/1012	26/26/18987	31/31/16849
	$C_{R}$	0.00%	42.90%	14.29%	95.35%	10%	96%	0.00%	0.00%	100%	100%
	$F_{R}$	0.00%	0.61%	0.09%	2.37%	0.13%	2.69%	0.00%	0.00%	0.14%	0.18%

**Table 3:** The quantitative evaluation results for the image matching.

Test Images	Metric	Original	Rectified	Rectified + Fusion
Pair 1	$N_{c}/N_{T}/Min\left(N_{L},N_{R}\right)$	186/257/22239	390/390/22239	527/527/22239
	$C_{R}$	72.37%	100%	100%
	$F_{R}$	0.84%	1.75%	2.37%
Pair 2	$N_{c}/N_{T}/Min\left(N_{L},N_{R}\right)$	163/269/24353	290/290/24353	439/439/24353
	$C_{R}$	60.59%	100%	100%
	$F_{R}$	0.67%	1.19%	1.80%
Pair 3	$N_{c}/N_{T}/Min\left(N_{L},N_{R}\right)$	752/752/25366	440/440/25366	1075/1075/25366
	$C_{R}$	100%	100%	100%
	$F_{R}$	2.96%	1.73%	4.24%
Pair 4	$N_{c}/N_{T}/Min\left(N_{L},N_{R}\right)$	339/364/24915	628/663/24915	839/858/24915
	$C_{R}$	93.13%	94.72%	97.79%
	$F_{R}$	1.36%	2.52%%	3.37%
Pair 5	$N_{c}/N_{T}/Min\left(N_{L},N_{R}\right)$	69/129/25034	452/573/20275	469/650/25034
	$C_{R}$	53.49%	78.88%%	72.15%
	$F_{R}$	0.26%	2.23%	1.87%

**Table 4:** The number of matches and the computation time for ASIFT, GMS, and the proposed method.

	Image Pairs		Pair 1	Pair 2	Pair 3	Pair 4
Metric						
Number of matches		ASIFT	170	273	33	92
		GMS	133	600	367	98
		Proposed	413	1187	895	438
Matching time (unit: s)		ASIFT	21.05	14.12	59.02	11.9
		GMS	1.36	2.30	6.32	1.19
		Proposed	11.07	11.25	18.12	7.53
